# *Borrelia crocidurae* Meningoencephalitis, West Africa

**DOI:** 10.3201/eid1902.121325

**Published:** 2013-02

**Authors:** Sandrine Goutier, Elisabeth Ferquel, Claudine Pinel, Annick Bosseray, Bruno Hoen, Gérard Couetdic, Amina Bourahoui, Claire Lapostolle, Hervé Pelloux, Martine Garnier, Natacha Sertour, Isabelle Pelloux, Patricia Pavese, Muriel Cornet

**Affiliations:** Author affiliations: Grenoble Teaching Hospital, Grenoble, France (S. Goutier, C. Pinel, A. Bosseray, H. Pelloux, I. Pelloux, P. Pavese, M. Cornet);; Pasteur Institute, Paris, France (E. Ferquel, M. Garnier, N. Sertour), Joseph Fourier University, Grenoble (C. Pinel, H. Pelloux, M. Cornet);; Besançon Teaching Hospital, Besançon, France (B. Hoen, G. Couetdic);; Argenteuil Hospital, Argenteuil, France (A. Bourahoui);; Laennec Hospital, Quimper, France (C. Lapostolle)

**Keywords:** *Borrelia crocidurae*, Borrelia meningitis, encephalitis, spirochete, molecular diagnosis, bacteria, tick-borne relapsing fever, Ornithodoros ticks, Lyme disease, West Africa

## Abstract

*Borrelia crocidurae*–associated relapsing fever is endemic to West Africa and is considered benign. We report 4 patients with *B. crocidurae*–associated neurologic symptoms; 2 of their cases had been misdiagnosed. Frequency and severity of this disease could be underestimated; molecular methods and serodiagnostic tests for Lyme disease might be helpful in its detection.

Tick-borne relapsing fever (TBRF) is caused by several *Borrelia* species that are transmitted through the bites of *Ornithodoros* ticks *(**1**).* TBRF is an acute febrile illness characterized by multiple recurrences of nonspecific signs and symptoms, including fever, headache, myalgia, and arthralgia. Neurologic complications might occur, particularly related to *B. hispanica, B. crocidurae, B. duttoni*, and *B. turicatae* infections *(**2**–**7**)*. Conventional diagnosis is made by microscopic detection of spirochetes in blood samples collected during acute febrile episodes and by direct examination of the cerebral spinal fluid (CSF) of patients with neurologic manifestations. Recently, molecular methods have been shown to be more reliable for *Borrelia* spp. detection in blood and CSF (*3*,*8*). *B. crocidurae* is endemic to West Africa; in Senegal, the rising incidence of infections reported recently has been associated with climate change (*1*,*9*). We report 3 cases of meningitis and 2 cases of encephalitis in 4 persons among a total of 11 consecutive travelers who returned from West Africa to France with *B. crocidurae* infections.

## The Study

Persons included in our study had clinical signs and symptoms of meningitis or encephalitis, or both, and were selected from the 11 patients with cases of *B. crocidurae* TBRF that were reported to and confirmed by the National Reference Center for Borrelia (NRCB) in France during 2009–2011. The NRCB is the reference laboratory responsible for the epidemiologic surveillance of TBRF in France. Clinical meningitis or encephalitis was defined as previously reported (*2**)*. *Borrelia* species were detected in Giemsa-stained thin blood smears by microscopy and quantitative buffy coat analysis (Becton Dickinson, Le Pont de Claix, France) when available *(**4**).* *Borrelia* spp. were detected and identified by using 16SrRNA PCR and subsequent sequencing as described (*8**)*. We tested serum and CSF samples with standardized antibody assays for detection of *Borrelia* spp. that cause Lyme disease (Table).

**Table:** Epidemiologic, clinical, and laboratory findings and treatment for 4 patients with *Borrelia crocidurae* meningitis, or encephalitis, or both*

Characteristic and treatment	Patient 1	Patient 2	Patient 3	Patient 4
Demographic factor				
Age, y/sex	36/M	57/M	7/F	26/M
Country of origin/of residence	Senegal/France	France/France	France/France	Senegal/France
Travel country	Senegal	Senegal	Senegal	Senegal
Travel dates	Mar–May 2009	May 2010	Feb–May 2011	Aug–Sep 2011
Travel duration, d	53	15	15	35
Travel accommodation	Family house	Hotel	Hotel	Family house
Arthropod or insect bite report	No	Yes	No	No
Individual vector protection	No	No	No	No
First suspected diagnosis†/presumptive treatment	Malaria/quinine	Sinusitis	Gastroenteritis	Malaria/piperaquine
Symptoms				
Oral temperature >38.5°C	Yes	Yes	Yes	Yes
Chills	No	No	Yes	Yes
Total no. febrile episodes/no. before diagnosis	2/1	4/2	6/5	2/1
Length of acute febrile episodes, d	2–8	2–6	2	2 to 6
Afebrile periods between febrile episodes, d	15	2–15	2–13	34
Asthenia/anorexia/weight loss	Yes/no/no	Yes/yes/yes‡	Yes/no/no	Yes/no/no
Headache	Yes (severe)	Yes	Yes (severe)	Yes (severe)
Myalgia	No	No	No	Yes
Photophobia, phonophobia	No	Yes	No	Yes
Neck stiffness	No	Yes	Yes	Yes
Cerebellar syndrome	No	No	No	Yes
Drowsiness	Yes	No	No	Yes
Imaging results				
Brain CT scan	Normal	Normal	ND	Normal
Brain MRI	Normal	ND	ND	Abnormal
Serologic results				
Leukocytes, g/L	4.7	15.8	13.0	8.4
Hemoglobin, g/L	134	139	115	131
Platelets, g/L	245	273	295	103
C-reactive protein, mg/L	103	4	57	150
Creatinine, μmol/L	107	76	42	99
*Borrelia* spp. detection, Giemsa-stained blood smear	+§	+§	+§	+¶
Quantitative buffy coat	ND	ND	ND	+
ELISA anti–*B. murgdorferi* (titer)				
Enzygnost Lyme IgG link VlsE Siemens#	+(11.3)	ND	ND	+(19)
Enzygnost Lyme IgM Siemens#	+(3.7)	ND	ND	+(1.21)
Western blot anti–*B. burgdorferi*				
Bioadvance IgG anti VlsE/p41/p83/p21**	+/−/–/+	ND	ND	ND
Euroimmun IgG anti–p17/p19/p21/p25/p30/p31/ p39/p83/VlsE††	ND	ND	ND	−/−/−/−/−/−/– /‡‡ /‡‡
Meridian IgM garinii/afzelii/p41/p39/p17	ND	ND	ND	ND
Bioadvance IgM anti p25/p83**	+/+	ND	ND	ND
16 rRNA PCR *Borrelia*/identification	+/*B. crocidurae*	+/*B. crocidurae*	+/*B. crocidurae*	+/*B. crocidurae*
CSF test results				
Leukocytes, cells/mm^3^	405	217	258	156
% Lymphocytes	94	80	90	84
Erythrocytes, cells/ mm^3^	0	7	12	5
Protein, g/L	0.66	1.38	0.39	0.44
Glucose, mmol/L	3.1	2.7	2.69	2.9
Chloride, mmol/L	114	113	117	115
Lactate, mmol/L	ND	ND	1.5	2.2
Direct examination (Gram stain)	–	–	–	–
Conventional bacterial culture	–	–	–	–
16 rRNA PCR *Borrelia/*Identification	+/*B. crocidurae*	+/*B. crocidurae*	+/*B. crocidurae*	–
ELISA anti-*B. burgdorferi*				
IgG enzygnost lyme IgG link VlsE Siemens	ND	ND	ND	+¶¶
IgM enzygnost lyme IgM Siemens	ND	ND	ND	–
Treatment (daily dose/total d)	Ceftriaxone (2 g/21)	Doxycycline (100 mg 2×d/10)	Ceftriaxone (2 g/14)	Doxycycline (100 mg 2×d/21), ceftriaxone (2 g/15)

*CT, computed tomography; MRI, magnetic resonance imaging; ND, not done; +, positive; –, negative; CSF, cerebrospinal fluid. †First suspected diagnosis was not biologically confirmed. ‡7 kg in 3 wk. §Second sample was positive. ¶First sample was positive after review prompted by the quantitative buffy coat result. #Siemens, Erlangen, Germany. **Bio-Advance, Bussy Saint Martin, France. ††Euroimmun Medizinische Labordiagnostika AG, Lübeck, Germany. ‡‡Ambiguous. §§Meridian Bioscience, Paris, France. ¶¶At 12-fold dilution (low level).

Among the 11 TBRF cases reported to NRCB during the 3-year study, we identified 4 (36%) cases of clinical meningitis or encephalitis, or both. The epidemiologic, clinical, and laboratory findings and the treatment of the 4 patients are documented in the Table. Three of the 4 patients were adult men, 26–57 years of age, and 1 was a 7-year-old girl. None of the patients were immunocompromised. They were all given appropriate antimalarial chemoprophylaxis. Patients 1 and 4 experienced their first febrile episode in Africa and were empirically treated with antimalarial drugs without biological confirmation of *Plasmodium* infection.

At the time of admission to health care facilities, all patients had fever and headache. Patients 2, 3, and 4 had signs of meningitis, including neck stiffness; patients 2 and 4 also had phonophobia and photophobia. Patients 1 and 4 had encephalitis with drowsiness which for patient 4 was accompanied by cerebellar syndrome (dysarthria and dysmetria). All patients except patient 3 underwent computed tomography scanning of the brain; no abnormality was detected. The 2 patients with encephalitis were examined by magnetic resonance imaging; in patient 4, a predominant positive contrast of the cerebellum leptomeninges on the right side was observed.

For all patients, 16S rRNA PCR and sequencing identified *B. crocidurae* (*8*) in blood samples. Laboratory analysis of the 4 CSF samples showed a lymphocytic pleocytosis, high protein concentrations, and a glucose value within reference range (Table). The molecular methods applied to CSF samples confirmed neurologic *B. crocidurae* infection in patients 1, 2, and 3. Serum samples collected from patients 1 and 4 at the time of diagnosis were tested by using Lyme disease serodiagnostic assays. ELISA detected substantial levels of IgM and IgG in samples from both patients; 1 was confirmed by Western blot analysis. The CSF sample from patient 4 showed a low level of IgG (Table).

All cases were treated with doxycycline or ceftriaxone, or both (Table). In all patients, fever resolved within 3 days of the beginning of the appropriate treatment, and the outcomes were favorable. No Jarisch-Herxheimer reaction was observed.

*B. crocidurae*–associated TBRF is an emerging disease that is considered to be benign *(**1**,**9**).* However, the series of infections reported here suggest that severe neurologic complications, notably, meningitis and encephalitis, occur more frequently than previously thought and could be particularly common in travelers who acquired this infection in West Africa. For the patients we studied, the earliest neurologic signs occurred during the second febrile episode, confirming previous studies reporting the onset of neurologic complications after the first episode *(**2**)*. However, facial palsy, often considered to be among the main clinical signs and symptoms of neuroborreliosis caused by TBRF-associated *Borrelia* species, was not observed in these patients *(**2**).* A similar clinical manifestation described in a recent case report of *B. crocidurae* encephalitis is entirely consistent with our observations *(**3**).*

Functional and experimental studies have focused on the capacity of TBRF-associated *Borrelia* species to cross the blood–brain barrier and to persist in the brain *(2–7,10,11)*. These studies have established *B. crocidurae* as the most neurotropic species, an observation consistent with this and other case series and case reports. In animal models, this feature has been associated with the presence of vascular microemboli in the brain of infected animals and the particular ability of *B. crocidurae* to form and bind to erythrocyte rosettes, a phenomenon also involved in cerebral malaria pathogenesis. Erythrocyte aggregation might prevent host–pathogen interactions and thereby protect the spirochetes from the specific immune response *(**10**,**12**,**13**).*

The rather high frequency and severity of neurologic complications associated with *B. crocidurae* infection raise the problem of distinguishing it from cerebral malaria, because the areas of endemicity of these 2 diseases largely coincide *(**1**,**9**).* Indeed, relapsing fever is frequently misdiagnosed as malaria, as it was for 2 of the patients we studied, who were initially treated with antimalarial drugs *(**14**)*. In this context, quantitative buffy coat analysis that can effectively detect each pathogen in blood might be of particular interest *(**4**).* In addition, our study confirms the usefulness of molecular methods applied to blood and CSF samples to confirm *Borrelia* infection (*3*,*8**)*. The negative result obtained by PCR of CSF from patient 4 could have been the consequence of inappropriate storage of the sample at high room temperature for 72 hours before analysis.

Lyme disease serodiagnostic testing of serum and CSF samples might be helpful. Indeed, cross-reacting IgG and IgM were detected by ELISAs and in Western blot assays. Because Lyme disease is endemic to France, our results could have been caused by the actual detection of *B. burgdorferi* sensu lato antibodies, although none of the patients had a known history of Lyme disease.

No specific recommendations have been proposed for the treatment of patients with TBRF neuroborreliosis. Erythromycin and penicillin have been reported to be ineffective *(**5*,*6**)*. In our series, all patients were prescribed either ceftriaxone or doxycycline, or both (Table), resulting in successful treatment of the disease. Thus, from the literature and our own experience, we suggest that TBRF with neurologic involvement should be treated with ceftriaxone or doxycycline for at least 21 days.

## Conclusions

Our study highlights the frequent occurrence of meningitis or encephalitis in patients with *B. crocidurae* TBRF acquired in West Africa. The clinical and radiologic manifestations suggest that this infection could be more severe than previously thought. Consequently, travelers returning from West Africa with febrile neurologic disorders should be tested immediately for biological confirmation of *Borrelia* infection through blood and CSF analyses, including molecular methods.
